# Depot-specific adaption of adipose tissue for different exercise approaches in high-fat diet/streptozocin-induced diabetic mice

**DOI:** 10.3389/fphys.2023.1189528

**Published:** 2023-07-06

**Authors:** Yifan Guo, Qilong Zhang, Lifang Zheng, Jian Shou, Shuzhao Zhuang, Weihua Xiao, Peijie Chen

**Affiliations:** ^1^ Shanghai Key Lab of Human Performance, Shanghai University of Sport, Shanghai, China; ^2^ The Key Lab of Exercise and Health Sciences of Ministry of Education, Shanghai University of Sport, Shanghai, China; ^3^ College of Physical Education, Shanghai University, Shanghai, China; ^4^ School of Life Sciences and Biotechnology, Shanghai Jiao Tong University, Shanghai, China; ^5^ Institute for Physical Activity and Nutrition, School of Exercise and Nutrition Sciences, Deakin University, Geelong, VIC, Australia

**Keywords:** exercise intervention, mitochondria, white adipose tissue browning, brown adipose tissue, type 2 diabetes mellitus

## Abstract

**Background:** Adipose tissue pathology plays a crucial role in the pathogenesis of type 2 diabetes mellitus. Understanding the impact of exercise training on adipose tissue adaptation is of paramount importance in enhancing metabolic health. In this study, we aimed to investigate the effects of various exercise modalities on three distinct adipose tissue depots, namely, interscapular brown adipose tissue (iBAT), subcutaneous white adipose tissue (sWAT), and epididymal white adipose tissue (eWAT), in a murine model of diabetes.

**Methods:** Male C57BL/6J mice received a 12-week high-fat diet and a single injection of streptozotocin, followed by an 8-week exercise intervention. The exercise intervention included swimming, resistance training, aerobic exercise, and high-intensity interval training (HIIT).

**Results:** We found that exercise training reduced body weight and body fat percentage, diminished adipocyte size and increased the expression of mitochondria-related genes (PGC1, COX4, and COX8B) in three adipose tissue depots. The effects of exercise on inflammatory status include a reduction in crown-like structures and the expression of inflammatory factors, mainly in eWAT. Besides, exercise only induces the browning of sWAT, which may be related to the expression of the sympathetic marker tyrosine hydroxylase. Among the four forms of exercise, HIIT was the most effective in reducing body fat percentage, increasing muscle mass and reducing eWAT adipocyte size. The expression of oxidative phosphorylation and thermogenesis-related genes in sWAT and eWAT was highest in the HIIT group.

**Conclusion:** When targeting adipose tissue to improve diabetes, HIIT may offer superior benefits and thus represents a more advantageous choice.

## Introduction

Traditionally, adipose tissue has been divided into white adipose tissue (WAT) and brown adipose tissue (BAT) based on function and morphology. WAT is subdivided into visceral adipose tissue and subcutaneous adipose tissue based on anatomical location (Zwick et al., 2018). White adipocytes have large lipid droplets with a low mitochondrion content and their main function is to store energy as triglycerides within the lipid droplets, whereas brown adipocytes have small lipid droplets with a high mitochondrion content and their main function is to burn energy through non-shivering thermogenesis (Giralt and Villarroya, 2013). Recent studies have shown that WAT produces inducible thermogenic adipocytes to increase energy expenditure under certain conditions, such as exposure to cold (Lim et al., 2012), β-adrenergic agonists (He et al., 2019), or exercise (Mu et al., 2021). These cells are called beige adipocytes (also called “brite,” “brown-like,” or “inducible brown”) and the process of their formation is called WAT browning (Giralt and Villarroya, 2013). Activation of BAT and induction of browning of WAT can accelerate glycolipid uptake and reduce the demand for insulin secretion. This may be a new strategy to improve glycolipid metabolism and insulin resistance in obese and type 2 diabetes mellitus (T2DM) patients (Cheng et al., 2021).

It is well-known that obesity, which is defined by an excessive white adipose tissue mass, is one of the main risk factors for insulin resistance and T2DM. Obesity leads to lesions in adipose tissue, including inflammation and insulin resistance (Kohlgruber and Lynch, 2015), and these changes are associated with adipocyte hypertrophy and are potential causes and contributors to the development of T2DM (Burhans et al., 2018). The adipocyte size was positively correlated with HbA1c, HOMA-IR, LDL, total cholesterol and triglycerides (Monickaraj et al., 2012). Adipocyte size is associated with metabolic dysfunction and may be a determinant of adipose insulin resistance. Systemic energy dysregulation and insulin resistance may also be affected by mitochondrial dysfunction in adipocytes. Mitochondria fuel oxidative phosphorylation by consuming oxygen at cytochrome c oxidase, which drives glucose uptake (Lewis et al., 2019). Obesity and T2DM lead to reduced mitochondrial content, decreased oxidative phosphorylation (OXPHOS) rate and excessive production of reactive oxygen species (Sharma, 2015; Wada and Nakatsuka, 2016; Shou et al., 2020). In addition, studies on animal models and humans have reported that obesity is associated with changes in mitochondrial morphology, reduced mitochondrial content, adipose tissue dysfunction and lower oxygen consumption (Rong et al., 2007; Yin et al., 2014). Several studies have shown that mitochondria in adipose tissue are closely linked to significant adipocyte biology (e.g., adipogenesis, lipid metabolism and thermogenesis) (Boudina and Graham, 2014). Activated mitochondrial uncoupling protein 1 (UCP1) in BAT can uncouple the transport of electrons in the respiratory chain, thereby blocking the production of ATP and allowing energy to be dissipated in the form of heat (Zafrir, 2013). A comparable process occurs in beige adipocytes.

The health benefits of exercise are well-recognized and are observed across multiple organ systems, including adipose tissue (Lehnig and Stanford, 2018; Chow et al., 2022). Studies have reported that swimming (Ghiasi et al., 2015), resistance training (Yang et al., 2014), aerobic exercise (Motahari-Tabari et al., 2014) and high-intensity interval training (Wang et al., 2022) improve glucose control and insulin sensitivity. However, different exercise regimens caused different changes in adipose tissue. For example, aerobic and high-intensity interval training may be more effective than resistance training in reducing adiposity (Willis et al., 2012; Chang et al., 2021). Exercise-induced adaptive changes also varied between different adipose tissue depots. Therefore, in this study, we investigated how different types of exercise affect different adipose tissue depots in diabetic mice.

## Materials and methods

### Animals

4-week-old C57BL/6J male mice were purchased from the Model Animal Research Center of Nanjing University (Nan Jing, China). All mice were housed in the specific-pathogen-free grade animal room of the Shanghai University of Sport. The temperature of the animal room was controlled at 23–25°C and the mice were given free access to food and water under a 12-h light/dark cycle. All the animal protocols used were approved by the Ethics Review Committee for Animal Experimentation of the Shanghai University of Sport (Approval No. 2016006).

After 1 week of adaptation, mice were randomized into ND and HFD groups. The ND mice were fed normal chow (D12450J; 3.85 kcal/g, 10% kcal from fat, 20% kcal from protein, SYSE Ltd., Jiangsu, China) and the mice in the HFD group were fed a high-fat diet (D12492; 5.24 kcal/g, 60% kcal from fat, 20% kcal from protein, SYSE Ltd., Jiangsu, China). 12 weeks later, according to the previously published protocols (Obrosov et al., 2017; Zheng et al., 2020), HFD mice received a single intraperitoneal injection of streptozotocin (STZ, S0130, Sigma-Aldrich; Merck KGaA, Darmstadt, Germany) dissolved in citrate buffer (pH 4.4) at a dose of 100 mg/kg, while the control mice received the same volume of citrate buffer. On the seventh day after the STZ injection, fasting glucose tests were performed, and mice with a fasting blood glucose concentration >16.7 mmol/L were considered diabetic mice (48 of 60). Diabetic mice were divided into five groups: sedentary group, swimming exercise group, resistance exercise group, aerobic exercise group and high-intensity interval training (HIIT) exercise group. Sedentary mice fed with normal chow were used as a control group. Each group contains nine mice.

### Exercise protocols

**FIGURE 1:**
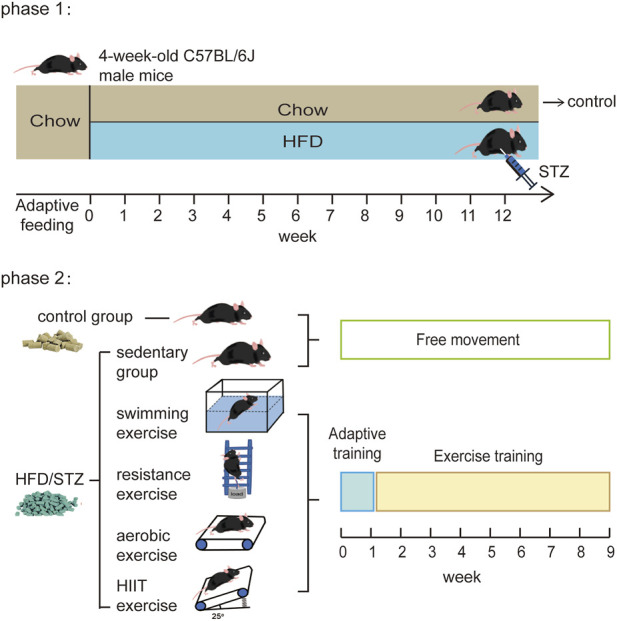
Experimental design diagram. In phase 2, the HFD/STZ mice were kept on a high-fat diet, and the control mice were kept on a normal diet.

The exercising mice received 9 weeks of different types of exercise training, including 1 week of adaptive training. During this process, the diet of each group of mice remains unchanged, with control mice receiving a normal diet and HFD/STZ group receiving a high-fat diet. The experimental design is shown in Figure 1.

The swimming training protocol was modified from a published article (Zhang et al., 2017). In a plastic container (60*50*50 cm) with a water depth of 45 cm and a temperature of approximately 35°C, the mice swam without any additional load. During the adaptive training, the mice swam gradually from 10 to 60 min per day, increasing the training time by 10 min per day. From the second week on, the mice were trained to swim without weights for 60 min per day, 5 days per week, for 8 weeks.

The resistance training protocol was based on published literature (Leite et al., 2013; Horii et al., 2018). A vertical ladder (1 m height, 2 cm intervals and 85° incline) was used to train the mice to climb the ladder with weight. On the first day of adaptation, the mice were trained to climb the ladder without load. The load was gradually increased each day until it reached 30% of body weight on the fifth day. From the second week onwards, the mice were in formal training with a load of 30% of their body weight. The training was performed 5 times per set (1 min interval between sets), 4 sets per day (2 min interval between sets), and training time was approximately 60 min per day. The training was carried out 5 days a week for 8 weeks. The load was gradually increased to reach 100% of the body weight by the 8th week of training.

Similar to other studies, the aerobic exercise protocol was performed on an animal treadmill. On the first day, training was started at a speed of 8 m/min for 10 min, and on the fifth day, the speed was increased to 15 m/min for 60 min. From the second week onwards, the speed was kept at 15 m/min and run for 60 min, 5 days a week for 8 weeks. Before each training session, mice run at 6 m/min for 5 min to warm up.

The protocol for HIIT was as previously described (Zheng et al., 2020). All mice in the HIIT group performed the exercise training program on a mouse treadmill at 25° inclination five times a week for 8 weeks. We gradually increased the incline and speed of the treadmill during adaptive training. During 8-week training, the mice started with a warm-up at 5 m/min for 10 min, in which the HIIT consisted of 10 rounds of 4 min of high-intensity treadmill running interspersed with 2 min of complete rest. The pace during HIIT increased gradually from 16 to 26 m/min over 8 weeks.

### Weight and body composition

Throughout the experiment, body weight was measured once a week. The body composition of the mice was assessed using the Echo-MRI system (Echo Medical Systems, Houston, TX) at the end of the experiment. The fat and lean mass measured by the MRI scans were normalized to body weight in terms of percentage fat mass and percentage lean mass.

### Histology

Adipose tissues were dissected and immediately fixed in 4% paraformaldehyde for 24 h at 4°C. Adipose tissues were then routinely processed for paraffin embedding, and 5 μm sections were cut and mounted on glass slides. The sections were deparaffinized and rehydrated using graded concentrations of ethanol in water. Subsequently, the sections were stained with hematoxylin-eosin (H&E) following a standard protocol. Six randomly selected fields per section were imaged by an Olympus microscope. Lipid droplet in BAT and white adipocyte area was determined and counted using ImageJ software (National Institutes of Health, Bethesda, MD, United States). At least 100 adipocytes were counted per field.

### Immunofluorescence staining

Tissues were embedded in paraffin, sectioned at 5 µm thickness, deparaffinized and rehydrated through graded concentrations of ethanol in water. Sections were then stained with primary antibodies for UCP1 (1:250, Cell Signaling Technology, Boston, MA, United States, 72,298) and TH (1:250, Cell Signaling Technology, Boston, MA, USA, 58,844) overnight at 4°C. The next day, tissue sections were washed in Tris-buffered saline plus 0.1% Tween 20 (TBST, 3 × 5 min) and then incubated with fluorochrome-conjugated secondary antibody (1:1000 in 1% BSA-TBST) for 1 h at room temperature. Then, the tissue sections were washed in TBST and nuclei were stained with DAPI. Images were acquired with a Zeiss LSM700 confocal microscope.

### RNA isolation and qRT-PCR analysis

Total RNA was isolated from adipose tissue using the RNAiso Plus Total RNA Kit (Takara Bio Inc., Shiga, Japan, 9109), and absorbance at 260 and 280 nm was measured by Thermo Scientific NanoDrop™ One Microvolume Spectrophotometers to determine its concentration and purity. 1 μg total RNA per sample was reverse-transcribed into cDNA using a commercial PrimeScript™ RT Master Mix (Takara Bio Inc., Shiga, Japan, RR036A) according to the manufacturer’s instructions. Real-time PCR was performed using ChamQ Universal SYBR qPCR Master Mix (Vazyme, Nanjing, China, Q711). Relative expression of the gene of interest was determined using β-actin as the housekeeping gene using the -ΔΔCT method. Sequences are shown in Table 1.

**TABLE 1:** Primers used for quantitative real-time PCR analysis.

Gene name	Forward primers	Reverse primers
MCP-1	TTA​AAA​ACC​TGG​ATC​GGA​ACC​AA	GCA​TTA​GCT​TCA​GAT​TTA​CGG​GT
IL-1β	GCA​ACT​GTT​CCT​GAA​CTC​AAC​T	ATC​TTT​TGG​GGT​CCG​TCA​ACT
PGC1α	GAA​AGG​GCC​AAA​CAG​AGA​GA	GTA​AAT​CAC​ACG​GCG​CTC​TT
COX4	TGA​ATG​GAA​GAC​AGT​TGT​GGG	GAT​CGA​AAG​TAT​GAG​GGA​TGG​G
COX8B	TGT​GGG​GAT​CTC​AGC​CAT​AGT	AGT​GGG​CTA​AGA​CCC​ATC​CTG
UCP1	CTG​CCA​GGA​CAG​TAC​CCA​AG	TCA​GCT​GTT​CAA​AGC​ACA​CA
TMEM26	ACC​CTG​TCA​TCC​CAC​AGA​G	TGT​TTG​GTG​GAG​TCC​TAA​GGT​C
β-actin	CACTGTGCCCATCTACGA	TGATGTCACGCACGATTT

### Western blot analysis

Total protein was extracted from adipose tissue using radio immunoprecipitation assay lysis buffer (Beyotime, Shanghai, China, P0013B). According to the manufacturer’s instructions, the concentration was determined using the bicinchoninic acid protein concentration assay kit (Beyotime, Shanghai, China, P0010). About 30 μg of protein was separated using a 10% SDS-PAGE gel. After transferring the proteins to 0.45 μm polyvinylidene difluoride membranes (Millipore, Bedford, MA, United States, IPVH00010), the membrane was blocked with 5% skim milk powder diluted in TBST for 2 h at room temperature. The membrane was then trimmed into narrow strips based on the molecular weight of the protein of interest using a marker as a reference. An antibody to a single target protein is used to detect these bands. The membranes were incubated with UCP1 (1:1000, Cell Signaling Technology, 72,298), TH (1:1000, Cell Signaling Technology, 58,844) and HSP90 (1:500, Santa Cruz Biotechnology, Dallas, TX, United States, sc-13119), respectively, overnight at 4°C on a shaker. After being washed with TBST, the membranes were incubated with HRP-conjugated antibodies for 2 h at room temperature. After cleaning the membrane again with TBST, it was placed under a developer for enhanced chemiluminescence detection and photography. ImageJ software was used to calculate the grayscale values of the target proteins.

### Statistics

Data normality was assessed using the Shapiro-Wilk test. All data resulted to be normally distributed (*p* > 0.05). The data are presented as mean ± SD. Statistical analysis was performed using SPSS 25.0 software (International Business Machines Corporation, Armonk, NY, United States). The Student's t-test was used to compare between two groups, while one-way ANOVA was used to compare more than two different groups. The Least Significant Difference (LSD) *post hoc* test was used for data with homogeneous variances, and the Games-Howell multiple comparisons test was performed for data with non-homogeneous variances. A *p*-value <0.05 was considered statistically significant.

## Results

### The effects of exercise training on the body composition

**FIGURE 2:**
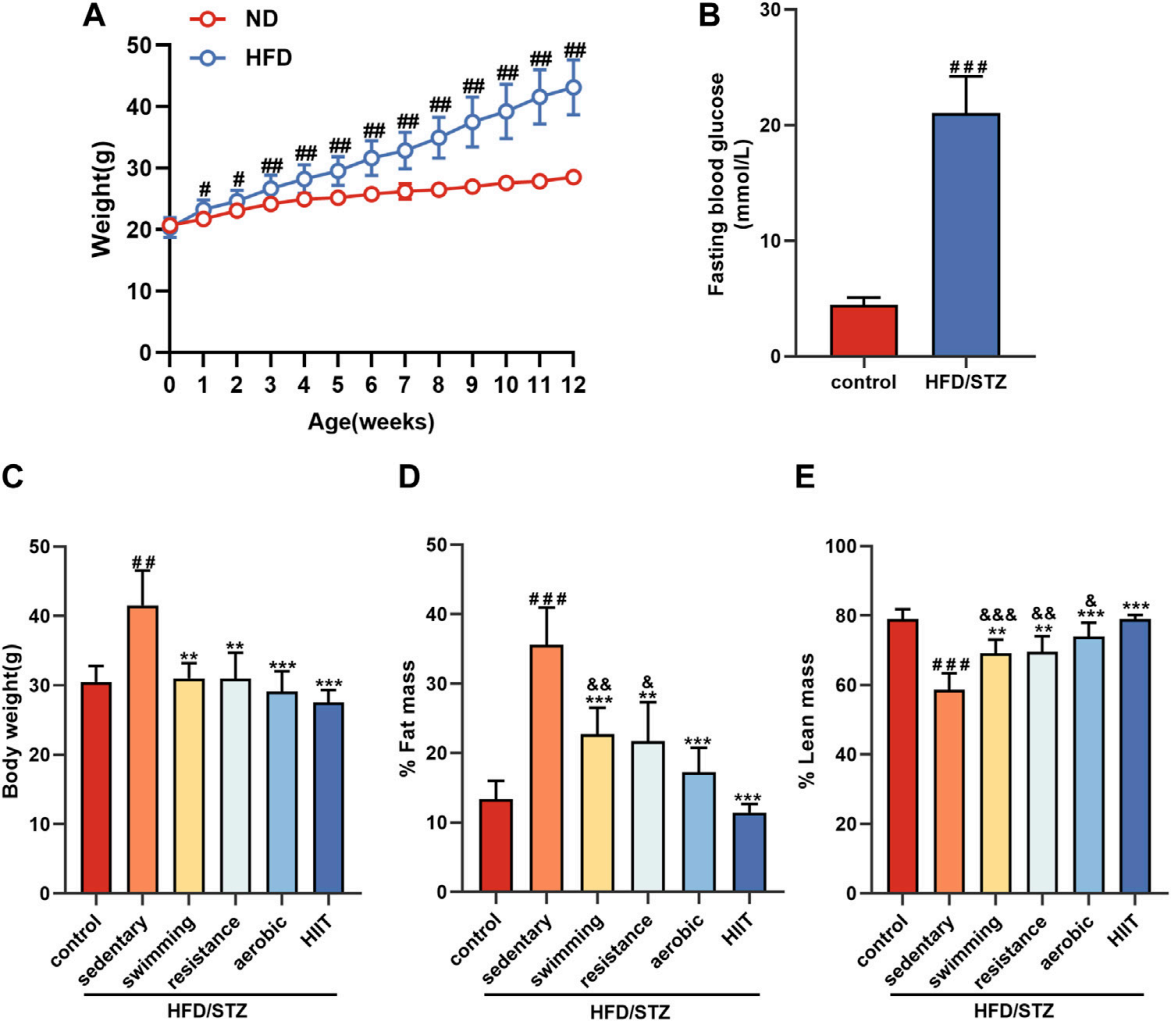
The effects of exercise training on the body composition **(A)** Weight change during 12 weeks of dietary intervention. **(B)** Fasting blood glucose was measured 1 week after 12 weeks of high-fat diet intervention followed by intraperitoneal injection of STZ. **(C–E)** Body composition of mice in control and HFD/STZ groups after exercise intervention. n = 9. All data are presented as mean ± SD. vs. control group: #*p* < 0.05, ##*p* < 0.01, ###*p* < 0.001; vs. sedentary group: **p < 0.01, ***p < 0.001; vs. HIIT group: &*p* < 0.05, &&*p* < 0.01, &&&*p* < 0.001.

After 12 weeks, mice in the high-fat diet (HFD) group weighed significantly more than mice fed normal chow (Figure 2A). After disruption of pancreatic β-cell function by injection of STZ into mice in the HFD group, fasting blood glucose was significantly elevated (Figure 2B). On this basis, we randomly divided the HFD/STZ mice with fasting glucose >16.7 mmol/L into a resting control group and four exercise groups (swimming, resistance exercise, aerobic exercise and HIIT). The echo-MRI results showed that HFD/STZ resulted in increased body weight and body fat percentage in mice, while all types of exercise reduced body weight, decreased body fat percentage and increased lean body mass content (Figures 2C–E). Notably, the HIIT group had the lowest body fat percentage and the highest percentage of lean body mass.

### The effects of exercise training on adipose tissue morphology and inflammatory factors

**FIGURE 3:**
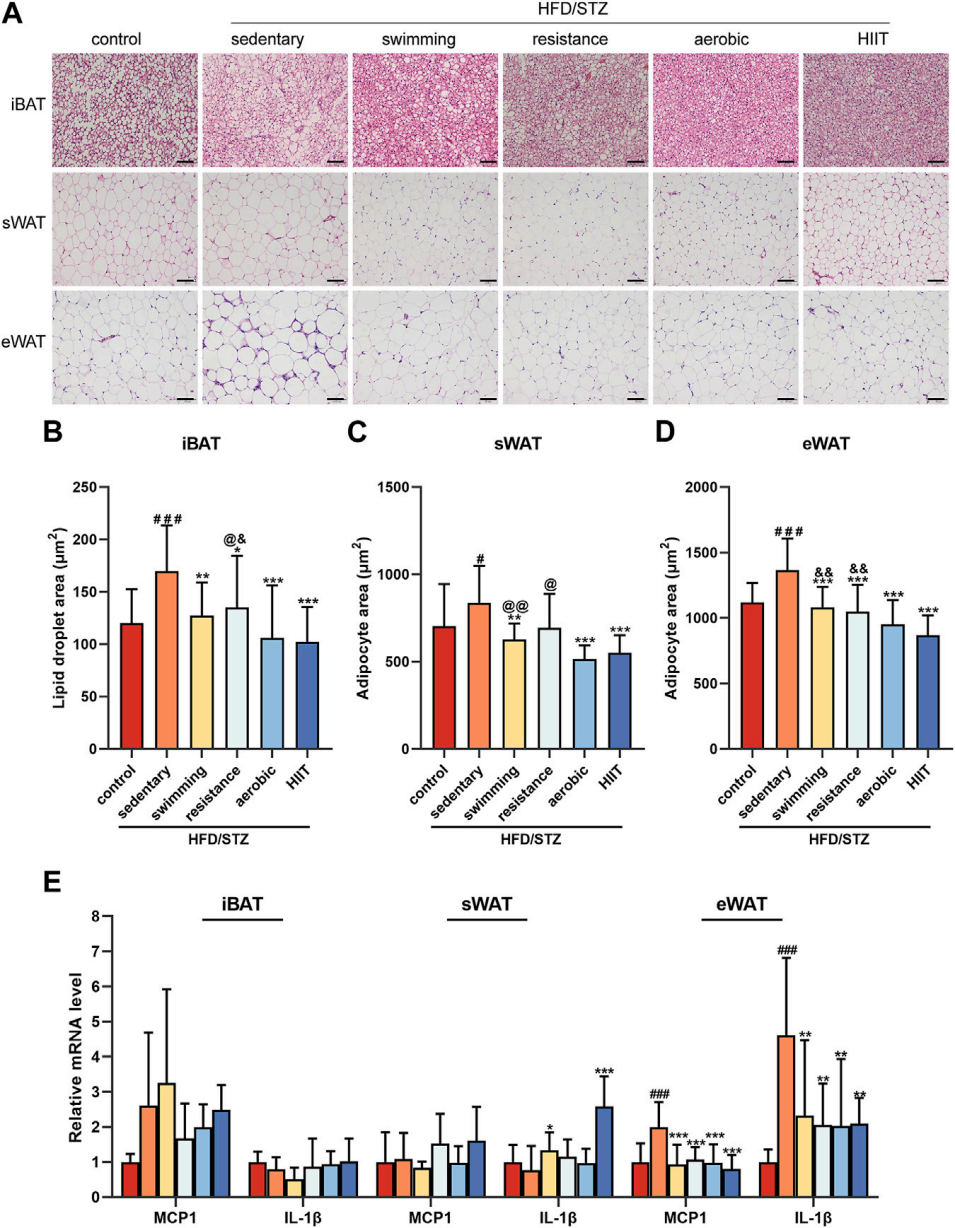
The effects of exercise training on adipose tissue morphology and inflammatory factors **(A)** H&E staining of iBAT, sWAT, and eWAT from control and HFD/STZ mice (magnification: ×200, scale bar:50 μm). Quantification of lipid droplet size in iBAT **(B)** and adipocyte size in sWAT **(C)** and eWAT **(D)**. Data were collected from H&E-stained sections of 3 individual mice in each group, 6 fields per mouse, measured by ImageJ software. **(E)** MCP1 and IL-1β gene expression in iBAT, sWAT and eWAT of control mice and HFD/STZ mice (n = 6 for each group). All data are presented as mean ± SD. vs. control group: ^#^ *p* < 0.05, ^###^ *p* < 0.001; vs. sedentary group: **p < 0.01, ***p < 0.001; vs. aerobic group: @*p* < 0.05, @@*p* < 0.01; vs. HIIT group: &*p* < 0.05, &&*p* < 0.01.

To observe the effect of exercise on the morphology of adipose tissue, we performed HE staining of interscapular brown adipose tissue (iBAT), inguinal subcutaneous white adipose tissue (sWAT) and epididymal white adipose tissue (eWAT) in mice. In all adipose depots of sedentary HFD/STZ mice, the adipocyte area was significantly enlarged. The results showed a significant reduction in adipocyte size in the sWAT and eWAT of mice in the exercise group, as well as a noticeable reduction in large lipid droplets in the iBAT (Figure 3A). Among the mice that received the exercise intervention, the aerobic and HIIT training groups exhibited smaller iBAT lipid droplet areas (Figure 3B), the aerobic group had the minimal adipocyte area in sWAT (Figure 3C), and the HIIT group had the smallest eWAT adipocyte area (Figure 3D). Notably, sedentary mice exhibited dense crown-like structures (CLS) in eWAT, while exercise significantly reduced CLS. We examined the expression of the inflammatory factors MCP1 and IL-1β. Consistently, all of the four exercise modalities significantly downregulated the expression of inflammatory factors in eWAT (Figure 3E). However, the expression of inflammatory factors was not altered in iBAT, and the mRNA level of IL-1β was significantly increased in sWAT in the swimming and HIIT groups compared to the sedentary group.

### The effects of exercise training on the expression of mitochondrial-related genes

**FIGURE 4:**
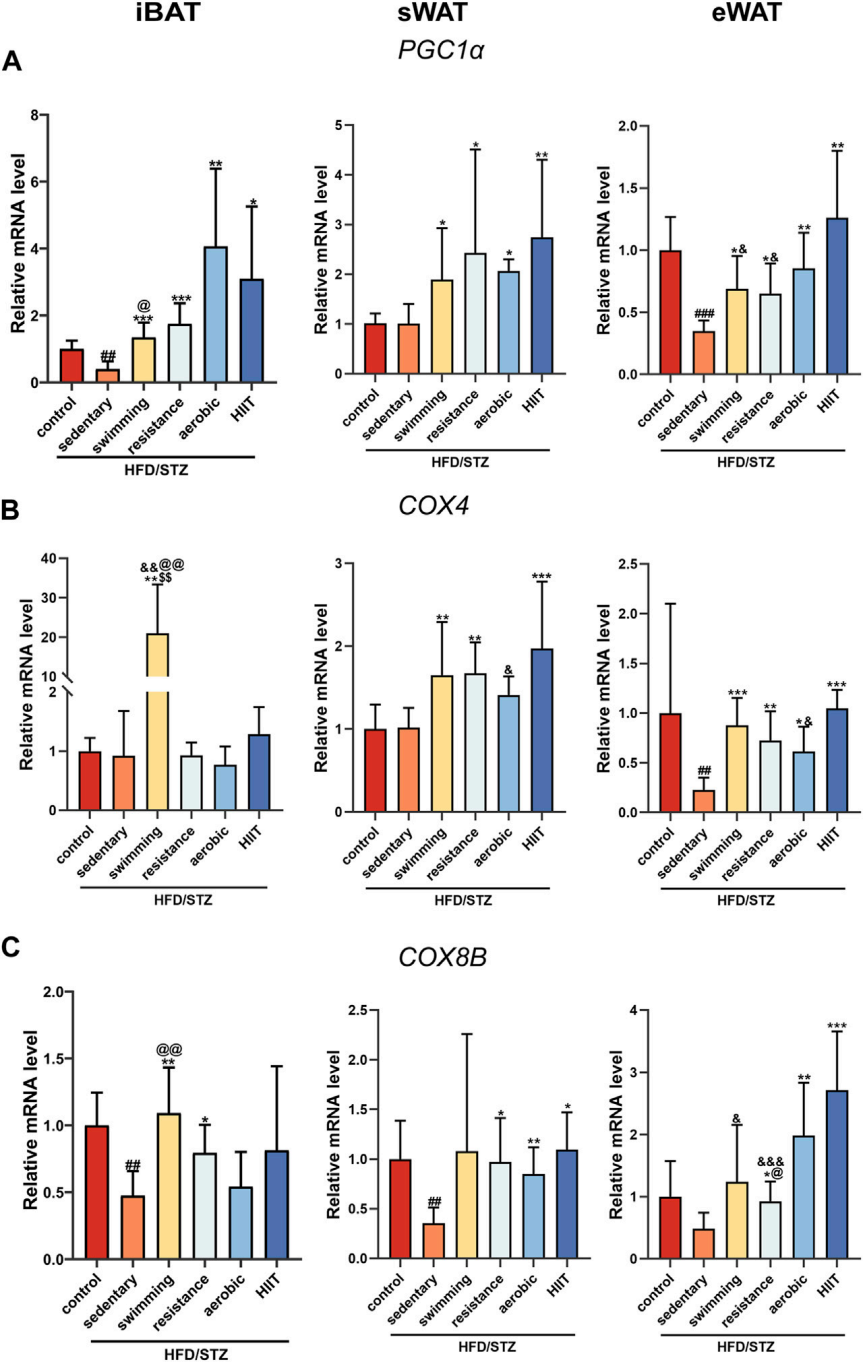
The effects of exercise training on the expression of mitochondrial-related genes. PGC1α **(A)**, COX4 **(B)** and COX8B **(C)** gene expression in iBAT, sWAT and eWAT of control mice and T2DM mice (n = 6 for each group). Values are mean ± SD and the expression of genes is corrected for the housekeeping gene β-actin. vs. control group: ^##^ *p* < 0.01, ^###^ *p* < 0.001; vs. sedentary group: *p < 0.05, **p < 0.01, ***p < 0.001; vs. resistance group: $$*p* < 0.01; vs. aerobic group: @*p* < 0.05, @@*p* < 0.01; vs. HIIT group: &*p* < 0.05, &&*p* < 0.01, &&&*p* < 0.001.

We examined the expression of mitochondria-related genes, due to the significant role that mitochondria play in adipose tissue remodeling, such as thermogenesis and browning of white adipose tissue (Zhu Q. et al., 2022). Peroxisome proliferator-activated receptor-γ coactivator-1α (PGC1α) is a co-transcriptional regulator that induces mitochondrial biogenesis through the activation of different transcription factors. Induction of PGC1α was associated with a consistent increase in mitochondrial content and drove the expression of many genes involved in mitochondrial OXPHOS, such as Cox8b and Cox4 (Puigserver and Spiegelman, 2003). In different depots of adipose tissue, HFD/STZ caused variable effects on mitochondria-related genes. The results showed that HFD/STZ reduced the expression of PGC1α and COX8B in iBAT, decreased the expression of COX8B in sWAT, and downregulated the PGC1α and COX4 expression in eWAT (Figures 4A–C). After 8 weeks of exercise training, the expression of PGC1α was markedly increased across all three depots (Figure 4A). In sWAT and eWAT, the four modalities of exercise intervention induced an upward trend in COX4 and COX8B mRNA levels, whereas in iBAT only resistance training increased COX4 and COX8B expression (Figures 4B,C).

### The effects of exercise training on UCP1 and TMEM26 mRNA level

**FIGURE 5:**
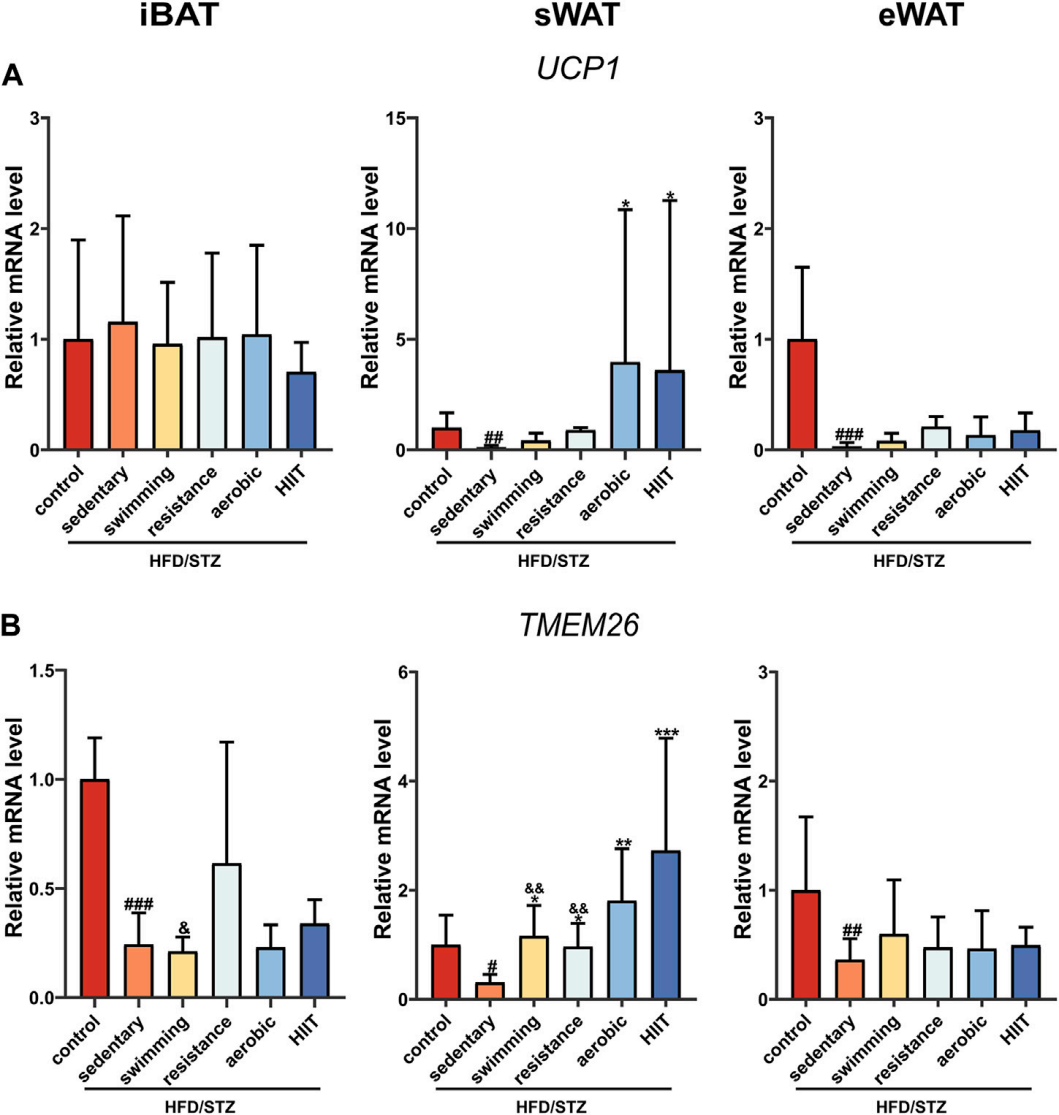
The effects of exercise training on UCP1 and TMEM26 mRNA level. UCP1 **(A)** and TEM2M26 **(B)** gene expression in iBAT, sWAT and eWAT of control mice and HFD/STZ mice (n = 6 for each group). Values are mean ± SD and the expression of genes is corrected for the housekeeping gene β-actin. vs. control group: ^#^ *p* < 0.05, ^##^ *p* < 0.01, ^###^ *p* < 0.001; vs. sedentary group: *p < 0.05, **p < 0.01, ***p < 0.001; vs. HIIT group: &*p* < 0.05, &&*p* < 0.01.

We examined mRNA levels of the key thermogenesis gene UCP1 and the brown/beige adipocyte-selected surface marker gene TMEM26. HFD/STZ significantly decreased UCP1 and TMEM26 expression in sWAT and eWAT, whereas only TMEM26 mRNA level was downregulated in iBAT. In sWAT, all four modalities of exercise upregulated TMEM26 expression, but only aerobic exercise and HIIT significantly induced an increase in UCP1 (Figures 5A,B). Notably, the expression of thermogenesis and beige cell-related genes was not altered in iBAT and eWAT in the exercise group.

### The effects of exercise training on UCP1 protein levels

**FIGURE 6:**
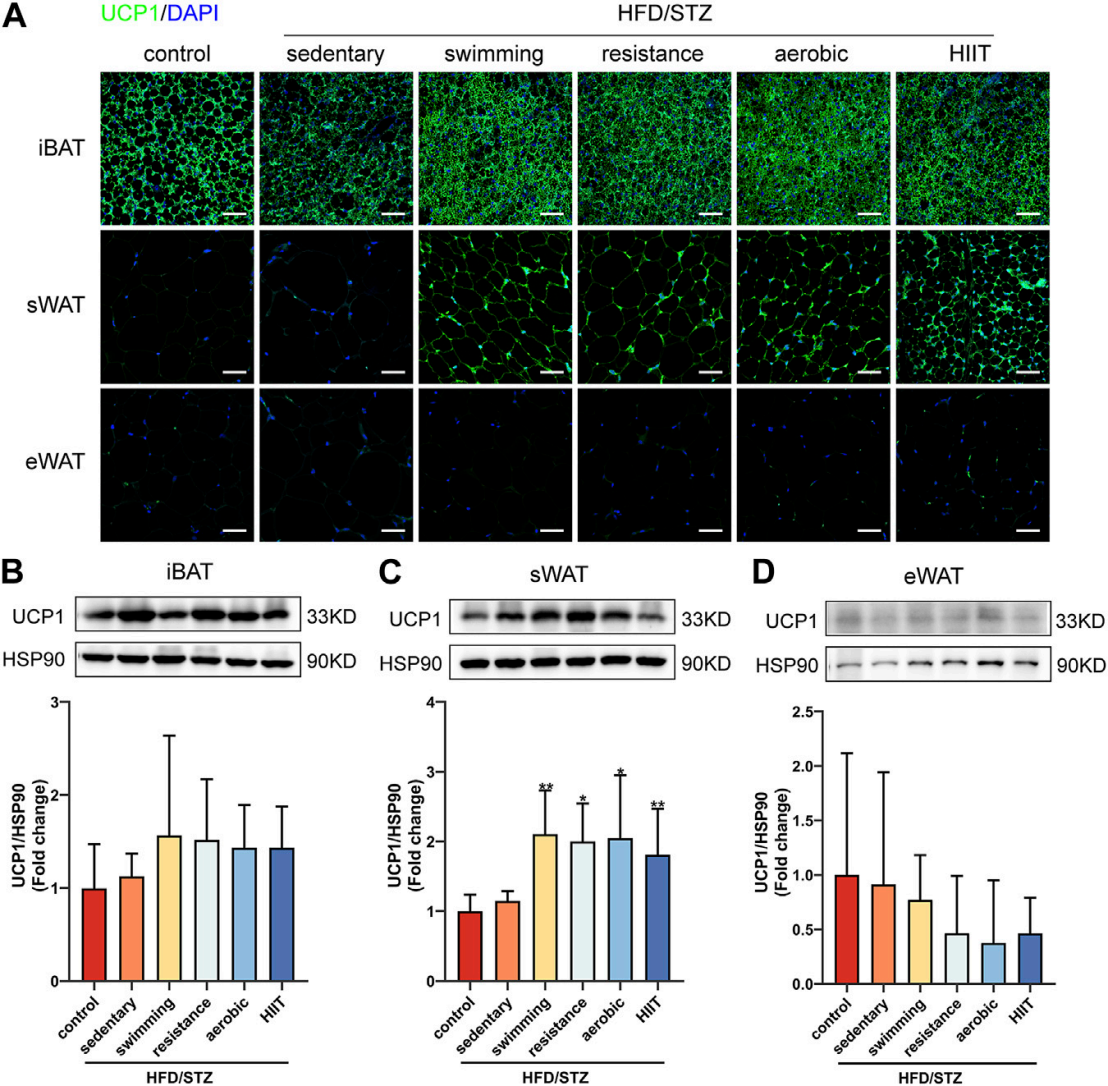
The effects of exercise training on UCP1 protein levels. **(A)** UCP1 immunofluorescence staining of iBAT, sWAT and eWAT from control and HFD/STZ mice (magnification: ×200, scale bar:50 μm). Western blot analysis and quantification for UCP1 using the total protein isolated from iBAT **(B)**, sWAT **(C)** and eWAT **(D)** for control and HFD/STZ mice. The protein content is expressed fold change to the control. All data are normalized to HSP90 and are expressed as mean ± SD (n = 6 for each group). vs. sedentary group: *p < 0.05, **p < 0.01.

To extend our observations at the genetic level, we examined the protein levels of UCP1 in adipose tissue. Regarding the effects of HFD/STZ, the observed changes in gene expression were not stretched to the protein level. In three depots of adipose tissue, HFD/STZ did not decrease UCP1 protein levels (Figures 6A–D). Exercise training significantly increased the presence of UCP1-positive multilocular adipocytes and UCP1 protein expression in sWAT, and there were no significant differences observed in UCP1 protein levels among the four exercise groups (Figures 6A,C). Consistent with mRNA levels, exercise did not alter UCP1 protein levels in iBAT and eWAT (Figures 6B,D).

### The effects of exercise training on TH-positive sympathetic nervous terminals

**FIGURE 7:**
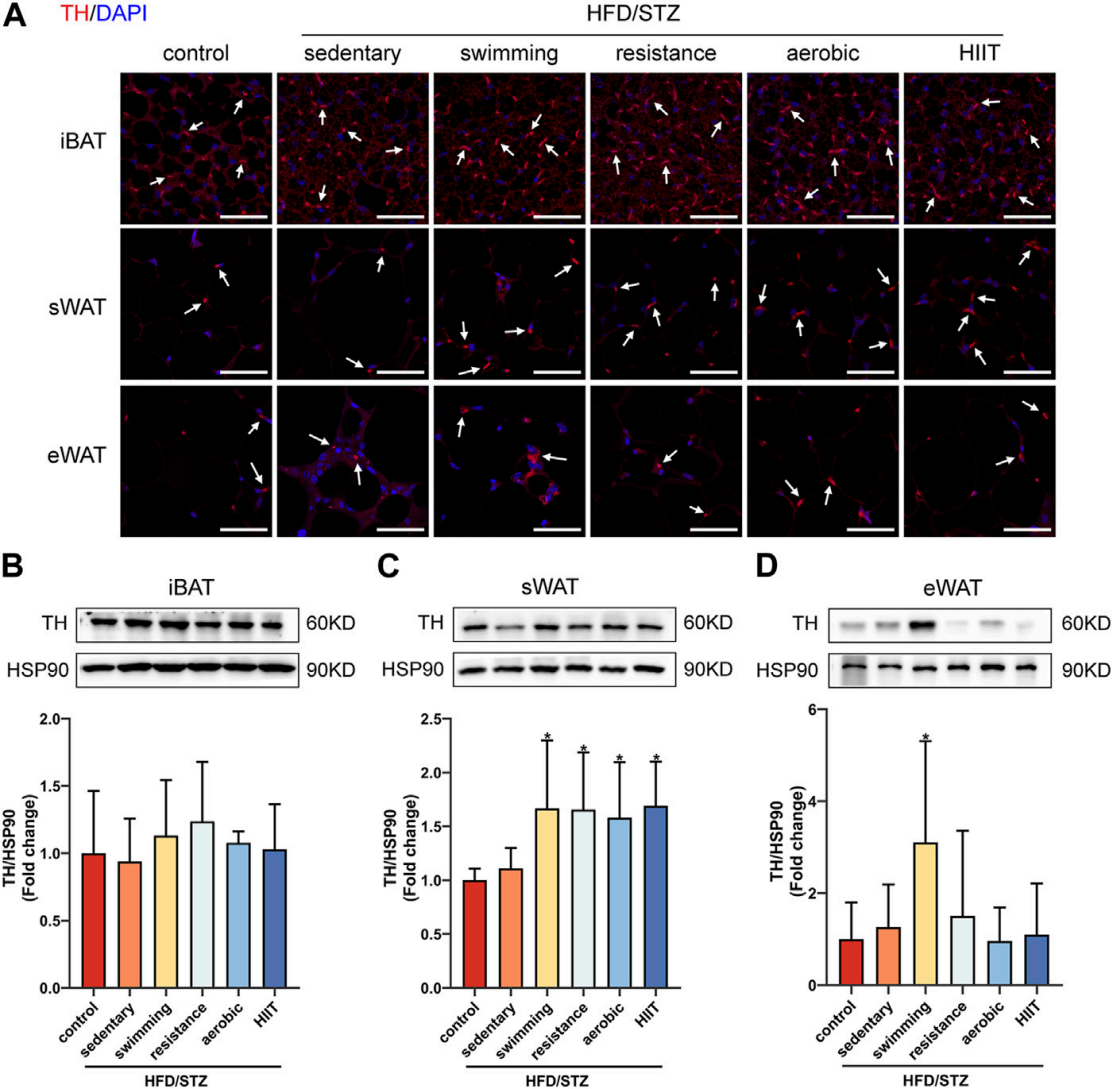
The effects of exercise training in TH-positive sympathetic nervous terminals. **(A)** TH immunofluorescence staining of iBAT, sWAT and eWAT from control and HFD/STZ mice, white arrows indicate TH-positive fibers (magnification: ×400, scale bar:50 μm). Western blot analysis and quantification for TH using the total protein isolated from iBAT **(B)**, sWAT **(C)** and eWAT **(D)** for control and HFD/STZ mice. The protein content is expressed relative to the control. All data are normalized to HSP90 and are expressed as mean ± SD (n = 6 for each group). *p < 0.05 vs. sedentary group.

We next explored the molecular mechanisms by which exercise training promotes sWAT browning in HFD/STZ mice. Since both thermogenesis and sWAT browning are regulated by the sympathetic nervous system, we compared the distribution of sympathetic neuron terminals in three depots of adipose tissue from control and HFD/STZ. A specific antibody against tyrosine hydroxylase (TH), a marker for sympathetic nervous terminals, was used for immunofluorescence staining. TH signal was most widespread and densely distributed in iBAT, whereas eWAT showed the least TH-positive staining (Figure 7A). We also measured the protein levels of TH in adipose tissue. There was no significant difference in protein expression of TH in the three adipose tissue depots between control mice and sedentary HFD/STZ mice (Figures 7B–D). Exercise training significantly upregulated TH protein expression in sWAT (Figure 7C). Notably, swimming training significantly upregulated the protein levels of TH in eWAT (Figure 7D).

## Discussion

The animal model of diabetes was established by a high-fat diet combined with STZ injection using C57BL/6J male mice. Swimming, resistance training, aerobic exercise and HIIT training were performed on HFD/STZ mice. We observed the effects of exercise in adipocyte size, inflammatory factor, mitochondrial function, thermogenic function and sympathetic nerves in iBAT, sWAT and eWAT.

Researchers initially used STZ to induce a stable model of type 1 diabetes. To achieve a slow pathogenesis of T2DM, young adult mice are fed a high-fat diet to elicit obesity and insulin resistance. Single or multiple injections with STZ then elicit partial loss of β-cells, which results in hyperinsulinemia and hyperglycemia (Kleinert et al., 2018). In our study, HFD combined with STZ injection caused a significant increase in fasting glucose. Our previous study also confirmed that mice suffer from glucose intolerance and insulin resistance through glucose and insulin tolerance tests. (Zheng et al., 2020). Weight loss is known to reverse the underlying metabolic abnormalities in T2DM, leading to improved glucose control. Adipose tissue pathology is a major potential driver of chronic diseases such as obesity and T2DM, and reducing adipose tissue in patients with T2DM may be beneficial, regardless of the baseline amount (Carrier, 2007; Lingvay et al., 2022). Our study found that exercise can also have many beneficial effects, such as reducing weight and fat mass, despite in the presence of sustained high-fat feeding.

Reduced adipocyte size is strongly associated with reduced body weight and body fat percentage (Larson-Meyer et al., 2006). Adipocyte size is a determinant of adipose tissue dysfunction and metabolic disease (Laforest et al., 2015; Stenkula and Erlanson-Albertsson, 2018; Li et al., 2023). Enlarged visceral abdominal adipocytes are a predictor of the prevalence of T2DM in the population (Weyer et al., 2000; Lönn et al., 2010; Anthanont et al., 2017). It has been reported that visceral and subcutaneous abdominal fat mass are positively associated with abdominal adipocyte size, whereas the relative amount of lower body fat mass is negatively associated with abdominal adipocyte size (Tchoukalova et al., 2008). Nonetheless, while dietary restriction did reduce body weight (by 10.4 kg), it did not change abdominal sWAT adipocyte size, which was decreased only with the addition of exercise (You et al., 2006). In the present study, we observed that HFD/STZ resulted in increased adipocyte size in sWAT and eWAT and larger lipid droplets in iBAT, whereas all four trainings reduced cell size. However, to clarify the mechanisms by which exercise reduces fat mass, further measurements of adipocyte proliferation are needed. In addition, we observed that exercise significantly reduced CLS and inflammatory factor expression in eWAT but not in sWAT and iBAT. This may contribute to the fact that visceral fat tends to have higher levels of macrophages, regulatory T cells, natural killer T cells and eosinophils compared to subcutaneous fat (Koenen et al., 2021).

Since mitochondria play an important role in the physiological function of adipose tissue, we investigated markers of mitochondrial biogenesis and function. Among these, PGC1α is a key transcriptional activator. Induction of PGC1α is associated with a sustained increase in mitochondrial content, which drives many genes involved in mitochondrial OXPHOS (COX8B, and COX4)(Gburcik et al., 2013). Our data show that T2DM leads to decreased expression of PGC1α in iBAT and eWAT, and also suppresses the expression of OXPHOS-related genes in three adipose tissues (Jeddi et al., 2021; Serdan et al., 2021). Earlier studies have reported the effect of exercise on adipose tissue mitochondria, with data showing that exercise increases the expression of PGC1α and OXPHOS-related genes (de Las Heras et al., 2018; Cao et al., 2021; Moslehi et al., 2021; Nederveen et al., 2021; Tanimura et al., 2022). However, these experiments were conducted in normal/obese mice rather than in diabetes mice. Compared to obese patients without diabetes, the abundance of several different classes of mitochondrial proteins is reduced in the adipose tissue of age and weight-matched T2DM patients (Carruthers et al., 2021). We observed that exercise significantly increased PGC1α expression in all three adipose tissue depots and upregulated COX8B and COX4 mRNA levels in sWAT and eWAT in HFD/STZ mice. In a study of healthy men with a family history of T2DM, it was shown that exercise increased the expression of genes involved in oxidative phosphorylation in sWAT(Rönn et al., 2014). Notably, in our study, the expression of OXPHOS-related genes in iBAT was increased only by swimming. Proteomics revealed that swimming exercise upregulated the iBAT OXPHOS pathway (Aldiss et al., 2020), but treadmill training did not alter BAT oxidative phosphorylation-related protein expression levels (Tsuzuki et al., 2022), which is consistent with our observations. Since there are studies demonstrating that even when the animals swim at warm water temperature (35–36°C), they lose body heat (Harri and Kuusela, 1986). The increase in OXPHOS of iBAT observed in the present study may be a response to the environment adaptation instead of exercise, since our animals may have a lower body temperature at the end of the swim while waiting for their fur to dry. It should be noted that mild-cold water (20°C) swimming does not exacerbate brown adipose tissue activation in mice (da Silva et al., 2020).

PGC1α is required for exercise to promote UCP1 upregulation in adipose tissue (Ringholm et al., 2013). Exercise increased PGC1α mRNA levels in iBAT, sWAT and eWAT, but upregulated UCP1 protein expression only in iWAT. Furthermore, only iWAT showed a significant upregulation of TMEM26, a specific marker for beige adipocytes. This suggests that different types of exercise training induce sWAT browning in HFD/STZ mice. Regarding the effect of exercise on iBAT thermogenesis, there are some conflicting results. An early study reported that long-term exercise training increased the mass and protein content of UCP1 in the BAT of mice (Oh-ishi et al., 1996) and enhanced BAT thermogenesis function (Wickler et al., 1987). In recent years, there have also been reports that acute exercise increases UCP1 expression in the BAT of obese mice (Gaspar et al., 2019). In contrast, 8 weeks of running on a treadmill or resistance training led to a significant decrease in the expression of UCP1 and PGC1α in the BAT of rats (Wu et al., 2014). Endurance training also inhibits the activity of the brown adipose tissue in men (Vosselman et al., 2015). Physiological differences between species, exercise modalities, timing, and intensity of exercise may account for these conflicting results. In our study, when intervention subjects and exercise duration were matched, swimming, resistance, aerobic, and HIIT training did not induce an upregulation of UCP1 expression in BAT. Therefore, we believe that the differences between the published results are more likely related to exercise intensity. As for eWAT, although exercise markedly enhanced the expression of mitochondria-related genes, it did not alter the expression of its beige adipocyte-associated markers. This is consistent with previous reports showing that 10 weeks of voluntary exercise on a treadmill promotes the expression of thermogenesis-related genes in sWAT but no significant changes in eWAT (Karadedeli et al., 2022). Endurance exercise and high-intensity training also did not affect the protein and gene expression of thermogenic markers in eWAT (Maharjan et al., 2021). Thus, exercise-induced browning of white adipose tissue occurs predominantly in sWAT (Zhu Y. et al., 2022).

These findings suggest that exercise-induced adaptations to iBAT, sWAT, and eWAT are different in mice, and we sought to explore the reasons for this depot-specific variability. The sympathetic nervous system may be one of the molecular mechanisms involved. External stimuli, such as exposure to cold, can activate sympathetic nerves to release catecholamine neurotransmitters (e.g., norepinephrine) to promote the formation of beige adipocytes in WAT and also activate BAT activity to increase energy expenditure to combat metabolism disorders (Bartness et al., 2010; Blaszkiewicz and Townsend, 2016). Few studies have addressed the sympathetic effects of exercise on adipose tissue. Exercise has been reported to increase mRNA levels of sympathetic markers in rat mesenteric fat (Mannerås et al., 2009), while moderate-intensity exercise increased sympathetic activity of the BAT in normal mice (Almeida et al., 2022). TH is a catecholamine synthase whose expression correlates with the rate of norepinephrine synthesis and sympathetic content (Giordano et al., 2005), and here we found a significant increase in TH expression in sWAT, but not in iBAT and eWAT, in HFD/STZ mice after exercise. These results support the hypothesis that the induction of sWAT browning by exercise training may be associated with a selective increase in the sympathetic drive.

Our study has several limitations. Exercise intensity may differ among the four exercise groups of mice and can be determined by blood lactate and maximal oxygen consumption tests. It is noteworthy that our mouse experiments were performed at 23–25°C rather than the thermoneutral zone of a mouse (−30°C) (Cannon and Nedergaard, 2011; Maloney et al., 2014). Mice are actually in a state of heat stress under such conditions, which may affect physiological activities such as thermogenesis, energy expenditure, and fat browning. Furthermore, it remains an open question whether exercise activates sympathetic activity in adipose tissue, as we did not measure catecholamine levels in the blood or adipose tissue.

## Conclusion

In this study, we found that HFD/STZ leads to increased body fat in mice, accompanied by adipocyte hypertrophy, increased inflammatory factors, impaired mitochondrial function and thermogenesis. Exercise training reduces body fat percentage, reduces cell size and inflammation, improves mitochondrial function and induces sWAT browning. The increased sympathetic drive may be associated with exercise-induced sWAT browning. These findings underscore the potential efficacy of exercise interventions, with a particular emphasis on HIIT, in ameliorating the metabolic health of individuals with diabetes through modulation of adipose tissue functionality.

## Data Availability

The original contributions presented in the study are included in the article/supplementary material, further inquiries can be directed to the corresponding authors.
